# A Quasi-Solid State DSSC with 10.1% Efficiency through Molecular Design of the Charge-Separation and -Transport

**DOI:** 10.1038/srep28022

**Published:** 2016-06-17

**Authors:** Michio Suzuka, Naoki Hayashi, Takashi Sekiguchi, Kouichi Sumioka, Masakazu Takata, Noriko Hayo, Hiroki Ikeda, Kenichi Oyaizu, Hiroyuki Nishide

**Affiliations:** 1 Department of Applied Chemistry, Waseda University, Tokyo 169-8555, Japan; 2 Material Research Laboratory, Panasonic Corporation, Osaka 571-8686, Japan; 3 Kyoto R&D Laboratory, Mitsubishi Paper Mills Limited, Kyoto 617-8666, Japan

## Abstract

Organic-based solar cells potentially offer a photovoltaic module with low production costs and low hazard risk of the components. We report organic dye-sensitized solar cells, fabricated with molecular designed indoline dyes in conjunction with highly reactive but robust nitroxide radical molecules as redox mediator in a quasi-solid gel form of the electrolyte. The cells achieve conversion efficiencies of 10.1% at 1 sun, and maintain the output performance even under interior lighting. The indoline dyes, customized by introducing long alkyl chains, specifically interact with the radical mediator to suppress a charge-recombination process at the dye interface. The radical mediator also facilitates the charge-transport with remarkably high electron self-exchange rate even in the quasi-solid state electrolyte to lead to a high fill factor.

Dye-sensitized solar cells (DSSCs) have received significant attention because of their ease and high reproducibility in wet-chemical fabrication, and various module types of DSSCs have already been examined in full-scale on windows and walls in the field^1^,^2^,^3^,^4^,^5^,^6^,^7^. Recent advances in the designing of dyes and electrolytes have improved power-conversion efficiency of cells up to 13%, where the records of high efficiency were achieved by modification of the dyes^8^,^9^ and a cobalt redox couple in acetonitrile electrolyte solution^10^. Quasi-solid state DSSCs have also been researched by using e.g. polymer gel electrolytes^11^, to give the cells less hazard or solvent spill-free property. However, they encountered the difficulty of high resistance in the mediator diffusion in the quasi-solid state gel electrolytes and tremendously suppressed cell efficiency. An organic-based and quasi-solid state DSSC would represent a way for handheld and indoor applications of photovoltaic cells.

Indolines (2,3-benzo-4,5-dihydroindoles) are typical organic dyes with high light-absorption and wide variety of possible derivatives. Mitsubishi Paper Mills Limited, the company that the authors (KS and MT) of this paper belong to, synthesized an array of indolines (D-coded) samples, which were tested as the dye sensitizers in DSSCs^12^,^13^,^14^. Previous modifications of the indoline skeleton, including works by the authors, suggested molecular design in terms of an extended π-conjugation, a combination of donor-acceptor moieties, and an anchoring group to the TiO_2_ nanoparticles of the photoanode. Some of the modified indoline derivatives, such as D205^15^, successfully enhanced the DSSC performance^12^. Note be taken here that the indoline dyes are significantly inexpensive dyes due to the very simple and easy synthetic route from abundant starting materials.

Recently, the authors at Waseda University (KO and HN) successfully utilized the redox-active radical molecule TEMPO (2,2,6,6-tetramethyl-piperidin *N*-oxyl) as an electro-active material and demonstrated a remarkably high current-density and power-rate capability in organic rechargeable batteries, of which test samples are on their way to be further investigated for their performance^16^,^17^,^18^. The power-rate performance was ascribed to very fast charge-propagation and -transport via the TEMPO radical derived from its reversible and rapid redox reactivity and electron self-exchange activity. The authors also demonstrated that this amazing reactivity of TEMPO was not spoiled in its quasi-solid states or polymeric analogues, i.e. both for TEMPO in polymer gels and TEMPO-containing polymers^18^. The quasi-solid state polymer gel led to obvious advantages for the battery, such as non-leakage of the electrolyte and long life-time^19^, which are foreseen to be the same advantages for DSSCs.

Radical-based redox mediators have been explored as an alternative to the iodide redox couple in DSSCs^20^,^21^,^22^,^23^. A high voltage of 0.8 V was reported for the DSSC based on combination of indoline and TEMPO^22^. It was then suggested that organic-based and highly reactive radical mediators are effective for organic dyes such as indoline dyes and not for standard metal complex dyes such as ruthenium(bipyridyl) derivatives, and that the redox potential or the energy level of the radical mediator could be matched to the HOMO level of dyes.

This paper describes chemical aspects of our successfully fabricated DSSCs, by designing the dye/mediator interaction for charge separation and the charge transfer with mediator molecule, to provide a totally organic-based and quasi-solid state photovoltaic power source.

## Results and Discussion

DSSCs were fabricated with designed indoline molecules as the dye sensitizer and with the polymer gels containing a highly reactive TEMPO radical/cation redox couple as the hole-transporting mediator and the optimized electrolyte (the composition in the Supplementary Information). The photoanode was prepared for efficient light-harvesting with the new dyes, a TiO_2_ nanoparticle-, and a light-scattering-layer (their thickness = 0.70 and 5.8 μm, respectively) atop an ATO substrate.

**Figure 1:**
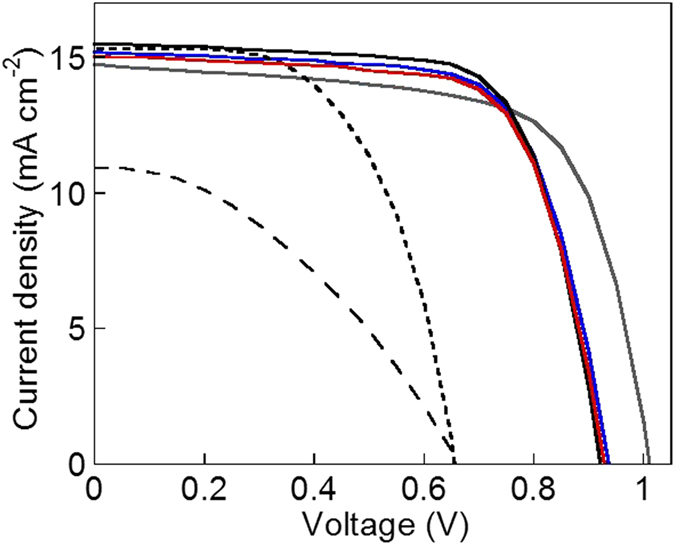
Photocurrent density-voltage (*J*-*V*) curves for the cells based on the TEMPO gel electrolyte. The cells sensitized with the MD-153 dye (red and blue curves), the ungelated TEMPO (black and gray curves), based on the ungelated iodide electrolyte (dotted curve), and the iodide gel (broken curve) with the optimized acetonitrile electrolyte (the details given in footnotes of Table 1).

The photocurrent density–voltage characteristics and the photovoltaic parameters of the cells are given in Fig. 1 and Table 1, along with those of the reference cells using the conventional iodide mediator and/or the commercially available indoline dye (D205) in almost identical electrolytes^12^. The combination of the long alkyl chains- and fluorene-derived indoline dye, the TEMPO mediator both in the gel and solution state, and tributyl phosphate^24^ successfully achieved a conversion efficiency (*η*) beyond 10%, which was reached through well-balanced and high short-circuit current density (*J*_sc_, above 15 mA cm^−2^), open-circuit voltage (*V*_oc_), and fill factor (*FF*). A large *V*_oc_ of almost 1 V could be ascribed to a more positive redox potential (in Table 2) or a deeper energy level of the TEMPO radical mediator in comparison with that of the iodide mediator^24^ (Diagram in Supplementary Figure S1). The high cell performance and the significantly high *FF* (above 0.7) were attributed to the low cell resistance resulting from the highly effective charge propagation and regeneration of the TEMPO mediator (described below using Table 2).

**Figure 2:**
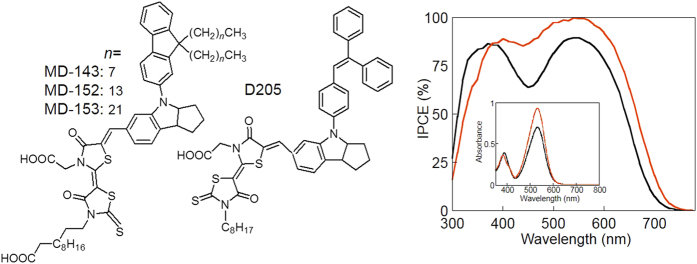
Molecular structures of the indoline dyes and IPCE. Normalized spectra for the optimized cells sensitized with the D205 (black curve) and the MD-153 (red curve) dyes and based on the TEMPO gel electrolyte. Inset: UV-vis spectra of 100 µM DMF solution of D205 (black curve) and MD-153 (red curve).

Typical examples of the indoline dyes as the sensitizer of DSSC, D205^12^ represented in Fig. 2, and its derivatives have been developed and commercially provided (the dye D series) by Mitsubishi Paper Mills. In this paper, the dyes were further developed along our three molecular design criteria: introducing a π-conjugated fluorene moiety, long alkyl chains and the second carboxylic acid on the dye. The carboxylic acid moiety in the dye acts as an anchor to bind on the TiO_2_ surface^25^. We anticipated that the thiazolidine skeleton worked as an electron-acceptor part in the dye (vs. the electron-donating indole residue) and that introduction of the second carboxylic acid on the thiazolidine could enhance the binding of the dye on TiO_2_. Binding of the new MD-dyes on TiO_2_ was facile and proceeded almost quantitatively by simply soaking the TiO_2_ nanoparticles in the *tert*-butyl alcohol/acetonitrile (1/1 = v/v) solutions of the dyes. The dye amount bound on TiO_2_ was analyzed by desorption treatment and e.g. found to be 2.5 × 10^−8^ mol cm^−2^, which was in the previously reported range^26^.

Fluorene is a well-known unit to enhance π-π^*^ absorption efficiency of its neighboring structures and is chemically robust even being exposed to redox reactions. The fluorene skeleton works also as a scaffold to introduce long alkyl chains; the alkyl chains have often been examined in the DSSC’s dyes to reduce the back electron-transfer or charge recombination at the dye/mediator interface^27^.

The re-designed and totally organic indoline-based dyes (coded MD-143, -152 and -153 in Fig. 2) were synthesized by reacting 4-[9,9-bis(*n*-alkyl)fluorene-2-yl]-1,2,3,3*a*,4,8*b*-hexahydrocyclopent[*b*]-indole-7-carbaldehyde and [3-(11-carboxyundecyl)4-oxo-2-thioxo-5-thiazolidinylidene]-4-oxo-3-thiazolidine acetic acid (Synthetic method and characterization were given in the Supplementary Information). The synthesis (simply heating in the acetic acid solution at 120 °C for 0.5 hr) and purification (recrystallization) of the dyes did not involve any expensive procedures or starting materials: Here being noted, the most effective dye (MD-153) bearing long C_22_H_45_ chains was synthesized using 1-docosanol, obtained from one of the most abundant fats, “Peanut Oil”. UV-vis absorption spectra of the dye solution given in Inset of Fig. 2 demonstrates very high light-absorption capability in the wavelength range of 350–700 nm (molecular absorption coefficient = 7.07 × 10^4^ M^−1^ cm^−1^ at the absorption maximum = 534 nm, slightly red-shifted in comparison with that of D205: 5.58 × 10^4^ M^−1 ^cm^−1^ and 530 nm, respectively^12^) originated from the combination of the indoline and the fluorene moieties. The visible absorption spectra were not changed after binding on the TiO_2_ particles, suggesting that the long alkyl chains did not influence the dye-binding process but rather suppressed aggregation of the dye while the classical indoline dyes are often *J*-aggregated with a broadened and red-shifted absorption. The incident photon-to-electron conversion efficiency (IPCE) spectrum of MD-153 (Fig. 2) coincided with its absorption maximum (Inset).

**Figure 3:**
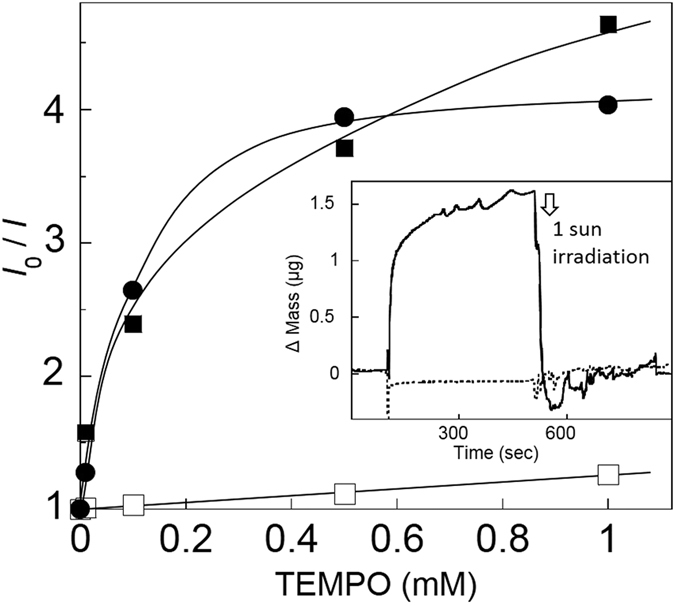
Stern-Volmer plots for the fluorescence quenching. TiO_2_ nanoparticles coated with the MD-153 dye dispersed in the acetonitrile electrolyte solutions with TEMPO as a quencher (closed circle plots), the MD-153 dye solubilized in the electrolyte with TEMPO (closed square plots), and the MD-153 dye with iodide (open square plots). *I* and *I*_*o*_: fluorescence intensity with and without a quencher, respectively. Inset: QCM adsorption profiles of MD-153 (solid curve) and D205 (dotted curve)-coated substrates in acetonitrile and 10 mM TEMPO under dark and 1 sun conditions.

HOMO levels of the dye molecules were estimated, using cyclic voltamograms of their solution, to be 1.08 V (vs. SHE) for the MD-dyes (1.06 V for D205), which was more positive or deeper (ca. 170 mV difference) than the redox potential of the TEMPO mediator at 0.91 V (see Table 2) and thus appropriate to reduce or regenerate the MD-dyes. The MD-indoline dyes including the ones adsorbed on TiO_2_ appeared bright red and emitted strong fluorescence at 640 nm upon irradiation (506 nm), which was efficiently quenched with the TEMPO radical mediator (TEMPO itself turned to the TEMPO cation). This reductive quenching was possible to monitor also for dispersions of MD-dye-bound TiO_2_ nanoparticles by carefully eliminating scattering noises, and was analyzed using the Stern-Volmer (S-V) equation (see the Supplementary Information). The S-V plots, with a normalized fluorescence intensity vs. concentration of TEMPO as a quencher, are given in Fig. 3 for the nanoparticle and the acetonitrile solutions. The plots followed a straight line (allowing calculation of the equilibrium constant (*K*_sv_) and the quenching rate constant (*k*_q_)) in a wide range of TEMPO concentrations for the reference D205 dye but only in the low TEMPO concentrations (followed quasi-straight lines) for the MD-dye. The significantly larger *K*_sv_ and *k*_q_ values for the MD-153 dye in comparison with those of the combinations of TEMPO/D205 and I_2_/MD-153 mean that TEMPO reductively regenerates the MD-153 dye very effectively even at the low TEMPO concentrations: [TEMPO]/[MD-153] = 0.1–1 (Supplementary Table S2). However, the S-V plots were deviating from a straight line or saturated at the high TEMPO concentration both for the MD-dye solution and the TiO_2_-bound MD-dye. These results suggested a specific and favorable interaction between the MD-153 dye and the TEMPO mediator, which helps explain the high *J*_sc_ and *η*.

Quartz crystal microbalance (QCM) offers an *in-situ* monitoring technique for adsorption of molecules in liquid-phase onto the QCM substrate^28^,^29^, and was applied to kinetically analyze an interaction of TEMPO in the electrolyte solution by using the substrates coated with the MD-153 and the D205 dye-bound TiO_2_ nanoparticles (Fig. 3, inset). The change of the quartz’s resonance frequency was semi-quantitatively correlated to the adsorbed mass on the substrates. The MD-153-bound QCM substrate was dipped in the acetonitrile electrolyte solution, and then TEMPO was injected in the solution. A fast and significant response of mass increase strongly suggested the adsorption of TEMPO, e.g. in Fig. 3 inset by 6.3 μg cm^−2^, which was not in conflict with the specific TEMPO uptake to MD-153 with [TEMPO]/[MD-153] = ca. 1. After the equilibrium was reached, 1 sun was momentarily irradiated, which led to an immediate loss of the increased or adsorption mass. On the other hand, similar experiments using/injecting the D205-bound substrate/TEMPO and the MD-153 substrate/iodide, respectively, did not cause any mass increase or loss that was detected from the noise in the experiment (i.e. any local temperature elevation upon the 1 sun irradiation).

**Figure 4:**
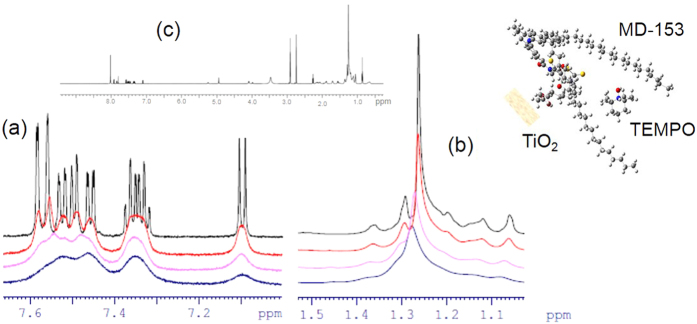
Paramagnetic shifts of NMR spectra. The spectra ascribed to (**a**) the aromatic rings and (**b**) the alkyl chains of the MD-153 dye of its 10 mM solution in the presence of 0 (black curve), 2 (red curve), 4 (pink curve), and 6 (blue curve) mM TEMPO mediator. (**c**) The overall NMR spectrum.

To further investigate the specific and favorable interaction of the new dye MD-153 and the TEMPO radical mediator, we monitored the paramagnetic shift in NMR spectroscopy on the dye molecule. TEMPO is an unpaired-electron bearing species and causes a proton NMR signals shift due to an external magnetic field-induced paramagnetic relaxation for a molecule^30^ which is located near the TEMPO. NMR spectra of the solution of MD-153 dye with the addition of TEMPO are shown in Fig. 4. The proton signals in the downfield, ascribed to the protons of aromatic rings of MD-153 (Fig. 4(a)), were simply broadened and not shifted with the TEMPO addition. Only the proton signals ascribed to the long alkyl chains of MD-153 around 1.25 ppm clearly decreased in intensity and shifted downfield with the added concentration of TEMPO (Fig. 4(b)), indicating an attracting interaction between the long C_22_ alkyl chains of the new dye and the TEMPO mediator. Additionally, this paramagnetic shift was rather enhanced at higher temperatures (Supplementary Figure S3), which suggests that the specific interaction could be classified into an entropic and de-solvation-related one, a solvophobic interaction.

Along the data of a paramagnetic shift in NMR and specific adsorption on QCM, we could conclude as follows. The indoline dyes exhibited a significantly high light absorption, and their derivatives, customized by introducing long alkyl chains (typically 22 carbon atoms), specifically interacted with the TEMPO mediator to reductively regenerate the dye, but did not interact with the corresponding TEMPO cation and thus suppressing a back electron-transfer process at the dye interface.

**Figure 5:**
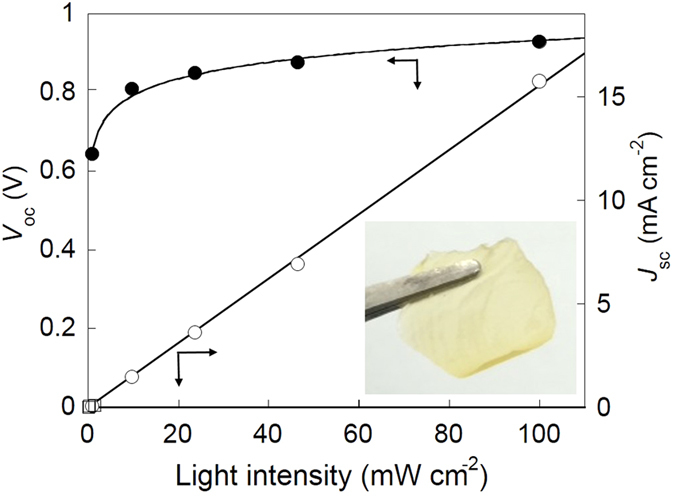
Irradiation light-intensity dependence of short-circuit current (*J*_sc_) (open plots) and open-circuit voltage (*V*_oc_) (closed plots). The cell sensitized with the MD-153 dye and based on TEMPO polymer gel electrolyte. Inset: a film of the quasi-solid state TEMPO gel electrolyte.

The *η *= 10.1% high performance of the MD-153 dye/TEMPO mediator/polymer gel-based DSSC cells was further investigated. Inset photo in Fig. 5 was exemplified the solvent elution-free, quasi-solid and flexible polymer gel electrolyte film, which displayed non-linkage and evaporation-loss of the electrolyte (given in the Supplementary Information S1). The TEMPO mediator worked even in the polymer gel very effectively, and its charge-propagation kinetic capability is given in Table 2 (Scheme for the mediation process with TEMPO/TEMPO cation redox couple in the DSSC is shown in Supplementary Figure S1). The heterogeneous electron-transfer rate constant (*k*_0_), estimated using the electrochemical Nicholson method, was 10^−1^ cm s^−1^ even in the quasi-solid state gel electrolyte and was 10 times larger than that of the I_2_ mediator. The diffusion coefficient (*D*_0_) of the TEMPO mediator, estimated with the electrochemical Cottrell method, in the gel was still very high at 3 × 10^−5^ cm^2 ^s^−1^, comparable to that in the corresponding homogeneous solution. The high reactivity of the TEMPO mediator in the gel was also analyzed using electron spin resonance (ESR) spectroscopy on the TEMPO and TEMPO cation mixture in the polymer gel electrolyte (Supplementary Figures S4 and S5). The electron self-exchange reaction or the charge-propagation rate constant (*k*_ex_) of TEMPO is given in Table 2. This very fast reactivity of TEMPO even in the quasi-solid state gel was reflected in the reduced resistance or enhanced *J*_sc_ and/or *FF* in the quasi-solid state DSSC cell system, which were in contrast to the significantly deteriorated performance for the I_2_ mediator/polymer gel-based cell (see Table 1). The highly diffusive TEMPO mediator reduced the possibility for a diffusion-limiting system, or avoided a mass-transfer limitation of the redox mediator often reported for the liquid DSSCs.

Figure 5 shows the light-intensity dependency of *J*_sc_ and *V*_oc_ of the MD-153/TEMPO polymer gel-based cell. Although *J*_sc_ linearly decreased with the light-intensity, *V*_oc_ maintained ca. 0.8 V even under low light-intensity of ca. 10 mW cm^−2^. This characteristic photovoltaic performance under low light intensity could be noted to be unique in comparison with the tremendous reduction of *V*_oc_ for amorphous silicon-based^31^ and organic photovoltaic cells^32^ (*V*_oc_ < 0.5 V) and even for the previous reported DSSC cell (*V*_oc_ < 0.8 V), which could be ascribed to the efficient charge-separation at the interface of MD-153 dye and TEMPO mediator described above.

## Conclusion

Organic-based solar cells that incorporate molecular-designed indoline dyes as sensitizer, in conjunction with highly reactive nitroxide radical TEMPO molecule as the redox mediator in polymer gel, displayed a well-balanced photovoltaic performance of high open-circuit voltage of 0.93 V, a high short-circuit current density of 15.5 mA/cm^2^, and a high fill factor of 0.70: Thus the cells achieved excellent conversion efficiency of 10.1%. The TEMPO mediator in the electrolyte, including the quasi-solid state form, possessed both appropriate redox potential and efficient reductive-quenching capability to regenerate the organic indoline molecule. It was also sufficiently kinetically active to facilitate charge-transport between the photoanode and the counter electrode with tremendously high values for diffusivity, heterogeneous electron-transfer rate, and electron self-exchanging reaction rate, which led to a very high fill factor and low cell resistance. We are now anticipating to replace acetonitrile with a non-volatile electrolyte solvent as such as imidazolium liquids^33^. The small decrease in output voltage of the cells even under low irradiation light intensities was described in this paper as an advantage of the organic-based cells. The cell is further developed by one of the authors’ group to facilitate the manufacturing of a totally organic-based, photovoltaic power source driven by interior lighting.

## Methods

The details of the fabrication of the DSSCs, the preparation of the MD-dyes and the gel electrolyte, and the characterization of the dyes, the mediator and the cells (NMR, ESR, fluorescence quenching, and *J*-*V* parameters) are described in the Supplementary Information.

## Additional Information

**How to cite this article**: Suzuka, M. *et al*. A Quasi-Solid State DSSC with 10.1% Efficiency through Molecular Design of the Charge-Separation and -Transport. *Sci. Rep*. **6**, 28022; doi: 10.1038/srep28022 (2016).

## Supplementary Material

Supplementary Information

## Figures and Tables

**Table 1:** Photovoltaic parameters, short-circuit current density (*J*_sc_), open-circuit voltage (*V*_oc_), fill factor (*FF*), and conversion efficiency (*η*) of the cells based on the TEMPO electrolytes.

Entry	Mediator	Dye	Electrolyte	*J*_SC_(mA cm^−2^)	*V*_OC_(V)	*FF*(−)	*η*(%)
1	TEMPO	MD-153	gel	15.5	0.93	0.70	10.1
2	TEMPO	MD-153	soln	15.7	0.95	0.69	10.4
3	TEMPO	MD-153	soln	15.1	1.02	0.64	9.9
4	TEMPO	MD-153	soln	14.0	0.89	0.71	8.9
5	TEMPO	MD-143	soln	13.4	0.78	0.74	7.8
6	TEMPO	MD-152	soln	14.3	0.82	0.76	8.8
7	TEMPO	D205	soln	9.9	0.88	0.75	6.5
8	iodide	MD-153	soln	13.5	0.72	0.42	4.0
9	Iodide	MD-153	gel	10.9	0.67	0.39	2.8

The cells sensitized with the MD-153 dye and based on the TEMPO polymer gel electrolyte, the TEMPO solution electrolyte, and based on the iodide electrolyte under 100 mW cm^−2^ full sunlight irradiation. The electrolyte was optimized with 1.5 M TEMPO, 0.025 M TEMPO cation, 1.2 M LiTFSI in acetonitrile, and 0.50 or 1.0 M tributyl phosphate was added for entry 3 and entry 1, 2 and 5–7, respectively. The gel or quasi-solid state electrolyte was prepared with poly(vinylidene fluoride-co-hexafluoropropylene).The standard deviation of *η* for entry 1 and 2 was 0.10 and 0.17%, respectively.

**Table 2:** Redox potential (*E*_1/2_), heterogeneous electron-transfer rate constant (*k*_0_), charge diffusion coefficient (*D*_0_), and electron self-exchange reaction rate constant (*k*_ex_) of the TEMPO- and iodide-based electrolyte in the gel state.

Mediator	*E*_1/2_ (V vs. SHE)	*k*_0_ (cm s^−1^)	*D*_0_ (10^−5^ cm^2 ^s^−1^)	*k*_ex_ (10^7^ M^−1 ^s^−1^)
TEMPO in gel	0.91	0.21	3.3	2.1
TEMPO	0.91	0.58	6.8	2.8
iodide in gel	0.65	0.017	0.25	–
iodide	0.65	0.023	1.2	–
