# Supplementary material for: A Quasi-Solid State DSSC with 10.1% Efficiency through Molecular Design of the Charge-Separation and -Transport
Source: Sci Rep. 2016 Jun 17;6:28022. doi: 10.1038/srep28022 (PMC4911559; doi:10.1038/srep28022)
Supplement: Supplementary Information [file srep28022-s1.pdf]

SUPPLEMENTARY INFORMATION

A Quasi-Solid State DSSC with 10.1% Efficiency through Molecular Design of the Charge-Separation and -Transport

Michio Suzuka[1,2], Naoki Hayashi[2], Takashi Sekiguchi[2], Kouichi Sumioka[3], Masakazu Takata[3], Noriko Hayo[1], Hiroki Ikeda[1], Kenichi Oyaizu[1], Hiroyuki Nishide[1*]

[1] Department of Applied Chemistry, Waseda University, Tokyo 169-8555, Japan

[2] Material Research Laboratory, Panasonic Corporation, Osaka 571-8686, Japan

[3] Kyoto R&D Laboratory, Mitsubishi Paper Mills Limited, Kyoto 617-8666, Japan

Contents

1. Additional Notes ········· 2
2. Materials and Methods ········· 4
3. Supporting Figures and Tables ········· 8

1. Additoinal Notes

Preparations of the gel electrolyte: The polymer gel electrolytes were prepared with 1.5 M TEMPO, 0.025 M $NOBF_4$, 0–1.0 M tributylphosphate, and 1.2 M lithium bis(trifluoromethanesulfonyl)imide dissolved in the acetonitrile solution of 10 wt% poly(vinylidene fluoride-*co*-hexafluoropropylene) (M_w ca. 4×10^5, Sigma-Aldrich Co.). The mixtures were heated at 80 °C for 6 hr to form the homogeneous solutions and then cooled down to r.t. for 3 hr to get the gels. The electrolytes for the experiment under interior lighting were prepared by replacing acetonitrile with 1-ethyl-3-methylimidazolium bis(trifluoromethanesulfonyl)imide (Tokyo Chemical Industry Co.) as the solvent.

Durability of MD-153: The new indoline MD-dyes were highly durable upon the photoredox processes. The reduction peak in cyclic voltammograms (ascribed to the radical cation state of the indoline dyes) was clearly observed in the repeated scanning.

Durability of the quasi-solid state gel electrolyte: The gel electrolytes were examined in stability or durability tests. Sandwiching the gel film with a pair of filter papers to squeeze out the electrolyte resulted in no significant mass decrease in the gel film and mass increase in the papers. The filter papers were subjected to ESR; the silent ESR supported that TEMPO was held in the gel. The gel electrolyte films were exposed to open air for 1 week: Around 20 wt% was lost for the gel film, but 96 wt% was lost for the ungelated film. The gel was composed of the imidazolium electrolyte, and none of the sealed cells exhibited any weight

loss in the tests.

Zeta potential of the MD-153 bound TiO_2 nanoparticles: We estimated the surface charge or zeta potential of the MD-153-bound TiO_2 nanoparticles in acetonitrile and the acetonitrile electrolyte solution to investigate the interaction of TEMPO mediators. The zeta potential values were 30 and 32 mV for the neat TiO_2 and the MD-153-bound TiO_2 in acetonitrile, respectively (43 mV in aqueous dispersion, as also has been reported in the previous paper: Boschloo, G., Haggman, L. & Hagfeldt, A. Quantification of the effect of 4-*tert*-butylpyridine addition to I^-/I_3^- redox electrolytes in dye-sensitized nanostructured TiO_2 solar cells. *J. Phys. Chem. B* 110, 13144–13150 (2006)). The zeta potentials indicated that the negatively charged surface of the TiO_2 nanoparticle was neutralized or rather slightly positively-charged after the bonding of the MD-153 dye in the solution including tributyl phosphate. Based on these results, we could explain the unexpectedly efficient dye regeneration or charge separation process, or minimized recombination in this DSSC with the specific interaction of the dye's long alkyl chains and with the TEMPO/TEMPO$^+$ redox couple, as represented below.

$$TiO_2/\text{MD-153/TEMPO} \xrightarrow{hv} TiO_2/\text{MD-153*/TEMPO}$$

$$\longrightarrow TiO_2(e^-)/\text{MD-153} + \text{TEMPO}^+$$

2. Materials and Methods

Materials: All starting reagents and solvents were purchased from commercial suppliers and used as received.

Cell fabrication: The working electrode was composed of a compact TiO_2/FTO (fluorine doped tin oxide) glass plate and a mesoporous TiO_2 layer adsorbed with dye molecules. The TiO_2/FTO glass was prepared by sputtering of a compact TiO_2 layer of 10 nm thicknesses on an FTO conductive glass plate (10 Ω/cm^2). The mesoporous TiO_2 layer (10 µm in thickness) was composed of a 0.9 µm absorption layer with TiO_2 particles (18 nm size) and a 9.1 µm scattering layer composed of TiO_2 particles (400 nm size) on the FTO plate. The layered mesoporous TiO_2 plate was immersed with 50 mM $TiCl_4$ aqueous solution at 70°C for 30 min, and rinsed with distilled water. The dye solutions were 0.3 mM in *tert*-butylalcohol/acetonitrile (v/v = 1/1). The FTO/mesoporous TiO_2 plate underwent dipping for 3 hr to complete the loading with dye sensitizer. The dye-adsorbed plate was washed with γ-butyrolactone for 3 min, followed by acetonitrile.

The counter electrode was prepared by depositing platinum black particles on an FTO glass plate (10 Ω/cm^2) with a doctor blade of a 5mM $H_2[PtCl_6]$ isopropyl alcohol solution and annealing at 450 °C for 30 min. The dried working electrode and the counter electrode were assembled using a 15 µm thick hot-melt polymer and heating at 100 °C. Action area of the

cells was 0.3 × 0.8 cm^2. The electrolyte was introduced into a predrilled hole and the hole was sealed by UV curing resin to yield quasi-solid state cells.

Measurements: Electrochemical analyses were carried out in a conventional cell under nitrogen. An ALS 660C electrochemical analyzer was employed to obtain cyclic voltammograms and chronopotentiograms.

A 450 W Xenon lamp and fluorescence lamp (Panasonic Co., FLR40S W/M) were used as the light source for the irradiation. *J–V* curves were recorded using a Keithley source meter. A silicon photodiode was used for the light intensity correction. The Keithley source meter (used in the *J–V* characterization) was utilized to measure the incident photon-to-collected electron conversion efficiency.

ESR spectra were recorded using a JEOL JES-TE200 spectrometer. The field modulation frequency and width were 100 kHz and 0.1 mT, respectively. ^{1}H NMR spectra were obtained using a Ultra Shield 600 MHz (Bruker Co.) spectrometer with chemical shifts downfield from tetramethylsilane as an internal standard. UV-Vis data for the dye solution were acquired with a F7000 UV-Vis spectrometer (Hitachi Co.).

QCM data were recorded using a crystal oscillator chemical measurement system (Seiko Co. QCA922) and an ALS 660DX electrochemical analyzer (BAS Inc.) with the fundamental frequency of the QCM (9.0 MHz), a platinum-sputtered titanium oxide QCM electrode (the

area 0.196 cm^2), and the shear modulus of quartz (2.95×10^{11} dyn cm^{-2}). The increment of the mass Δm accompanied by the reduction was determined by employing Sauerbrey's equation.

Fluorescence quenching measurement: Fluorescence spectra were measured using a spectrofluorophotometer (Hitachi Co., F-7000) with a 150 W xenon lamp as the excitation light source. Fluorescence quenching parameters between the dyes and TEMPO were estimated from the following equation:

$I_0/I = 1 + K_{sv}$ [Med] $= 1 + k_q t_0$

where K_{sv} is Stern-Volmer-equilibrium constant, I_0 is initial fluorescence intensity, I is fluorescence intensity, K_{sv} is Stern-Volmer constant, k_q is quenching rate constant, and t_0 is fluorescence lifetime. t_0 of the dyes was estimated to be 965 and 691 psec for MD-153 and D205, respectively, using a compact fluorescence lifetime spectrometer (Hamamatsu Photonics Co., C11367) upon excitation laser irradiation at 506 nm.

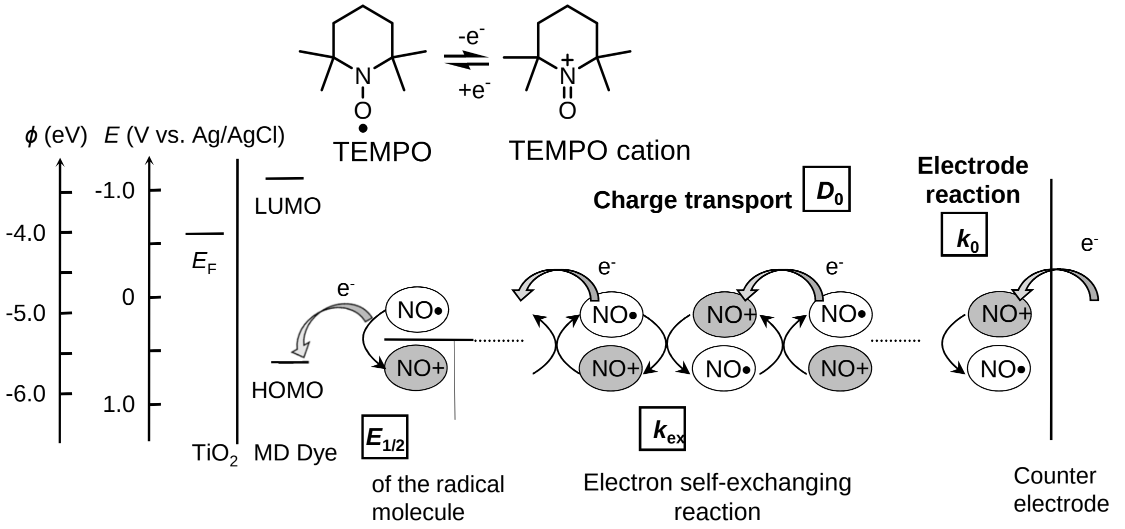

Figure S1 | Chemical structure of the TEMPO radical/cation redox couple and the charge-transport parameters in DSSC.

Table S1 | Optical and electrochemical properties of MD-153 dye.

dye	E_{ox} (V vs. Ag/AgCl)	λ_{max} (nm)	ε ($M^{-1}\ cm^{-1}$)	HOMO (eV)	LUMO (eV)
D205	0.85	530	53200	-5.51	-3.36
MD-153	0.86	534	70700	-5.52	-3.30

The λ_{max} and ε values were obtained from UV-vis spectra of 0.1 mM dye DMF solution. The HOMO level was estimated by CV. The LUMO level was calculated from the HOMO level and the absorption edge wavelength of UV-vis spectra.

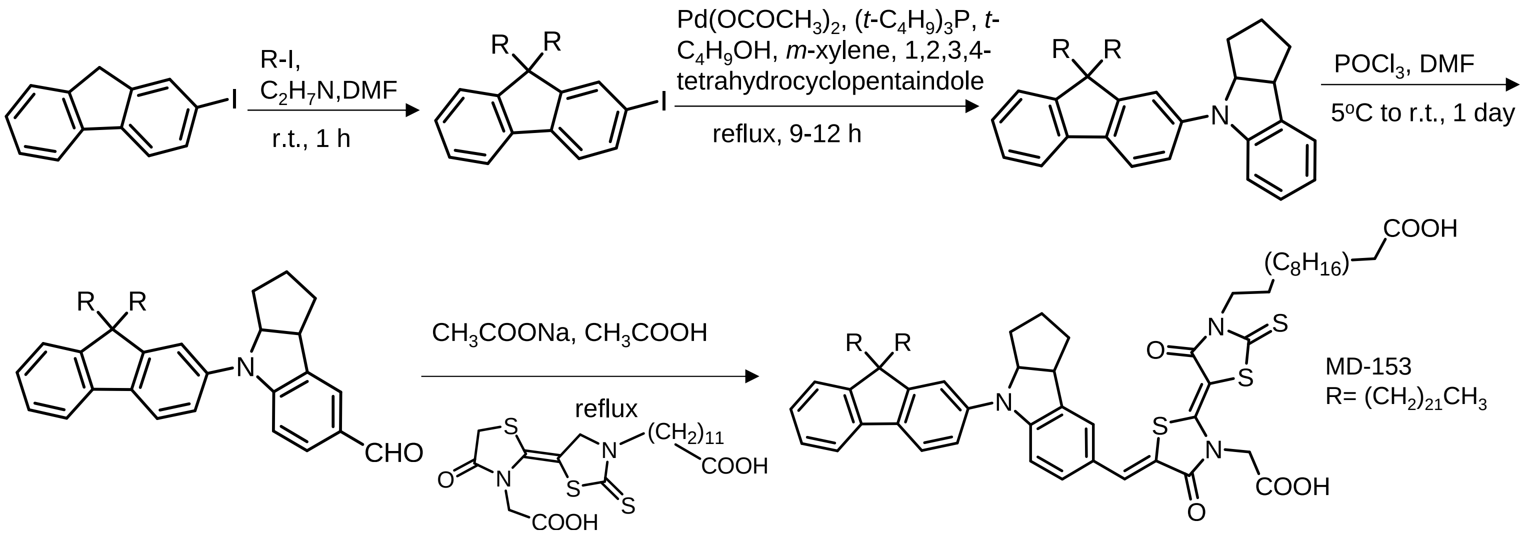

Figure S2 | Synthetic route of MD-153 dye.

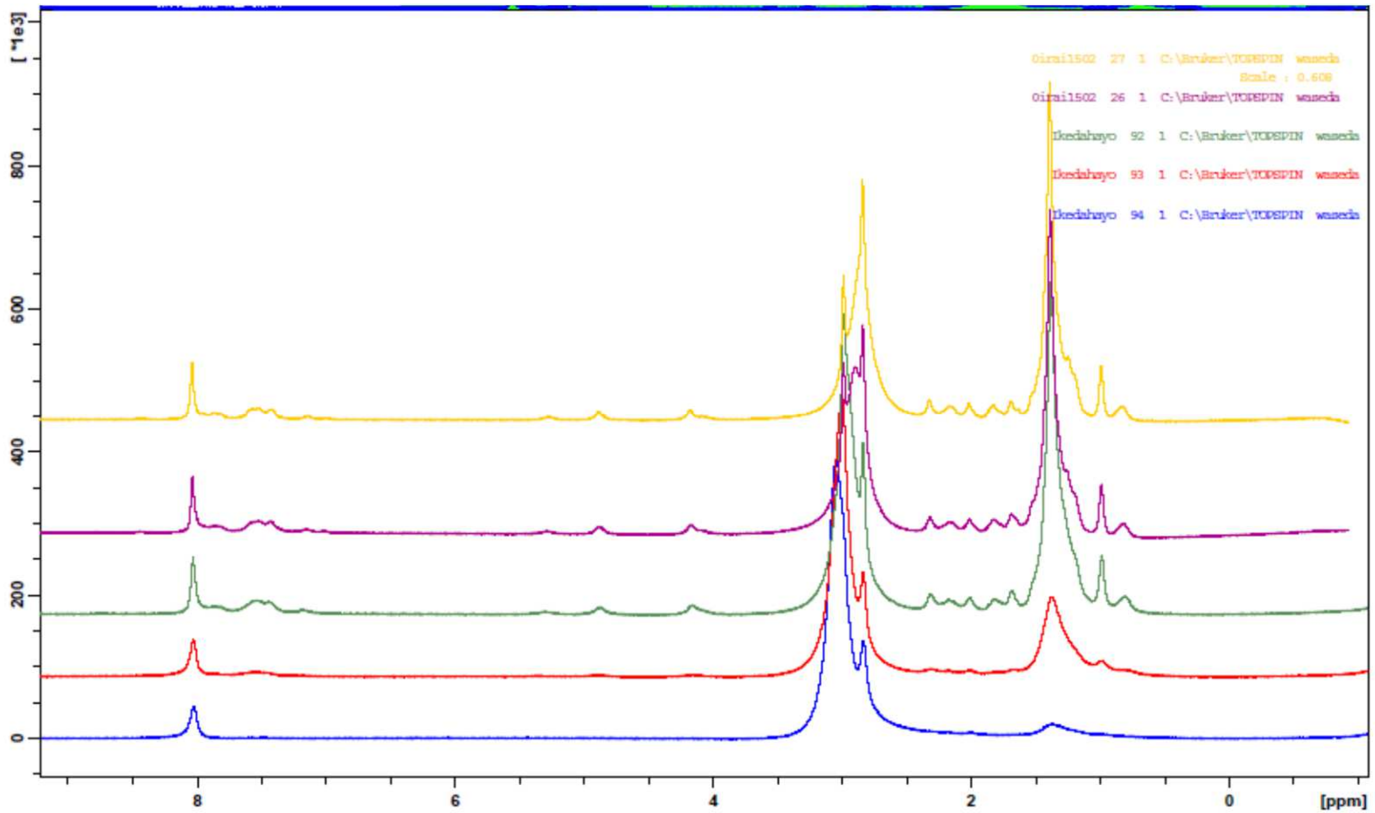

Figure S3 | Temperature dependence (5–50 ^{o}C) of the paramagnetic shifts of NMR spectra of the MD-153 dye in ^{d}DMF.

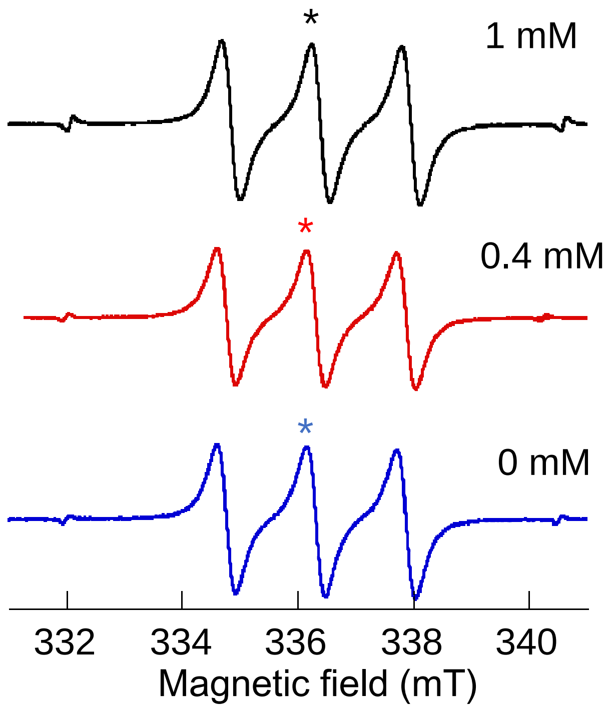

Figure S4 | ESR spectra of TEMPO. The spectra obtained for the gel of 1 mM TEMPO in the presence of 0, 0.4, and 1 mM TEMPO cation. Signals were calibrated on a Mn^{2+} standard.

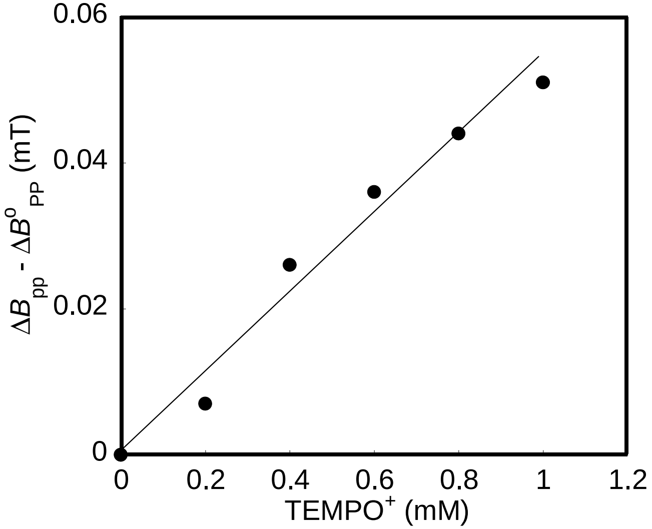

Figure S5 | Plots of the peak-to-peak line width for the ESR signal marked with an asterisk in Figure S4, vs the concentration of TEMPO cation.

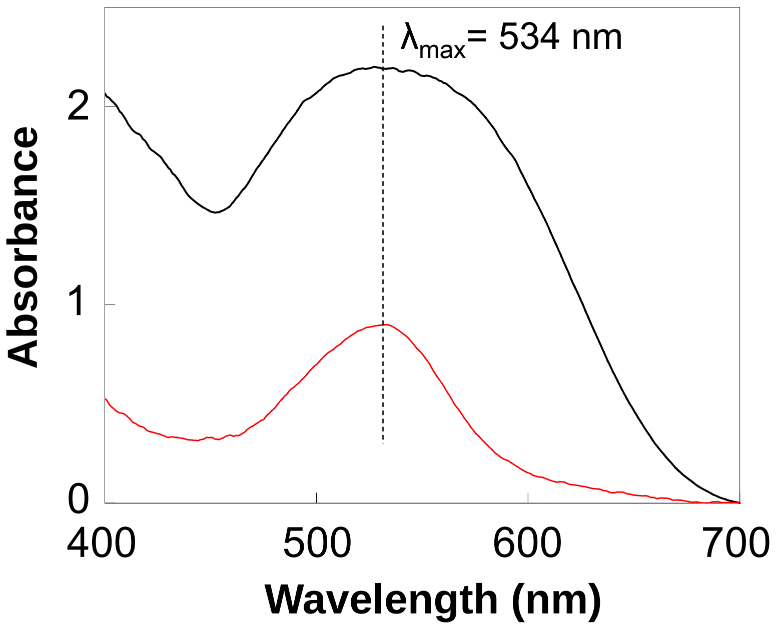

Figure S6 | UV-vis spectra of MD-153 in the 0.1 mM DMF solution (red curve) and adsorbed on TiO_2 particles (black curve).

Table S2 | Stern-Volmer-equilibrium constant (K_{sv}) and -rate constant (k_q) for the fluorescence quenching of the dyes absorbed on TiO_2 nanoparticle (dispersion) and in the acetonitrile solution

dye	mediator	K_{sv} (10^2 M^{-1})	k_q (10^{11} M^{-1} s^{-1})
MD-153/TiO_2	TEMPO	(1400)	(1400)
MD-153	TEMPO	(1200)	(1200)
D205	TEMPO	2.4	3.5
MD-153	Iodide	21	22

The dyes are reductively quenched with TEMPO or iodide. The parameters were estimated with the data in Fig. 3 with Stern-Volmer equation (linearly extrapolated for the MD-153 in the range of low TEMPO concentration 0–0.1 mM).
